# Supplementary figures and images for: Cancer care disruption and reorganisation during the COVID-19 pandemic in Australia: A patient, carer and healthcare worker perspective
Source: PLoS One. 2021 Sep 17;16(9):e0257420. doi: 10.1371/journal.pone.0257420 (PMC8448370; doi:10.1371/journal.pone.0257420)

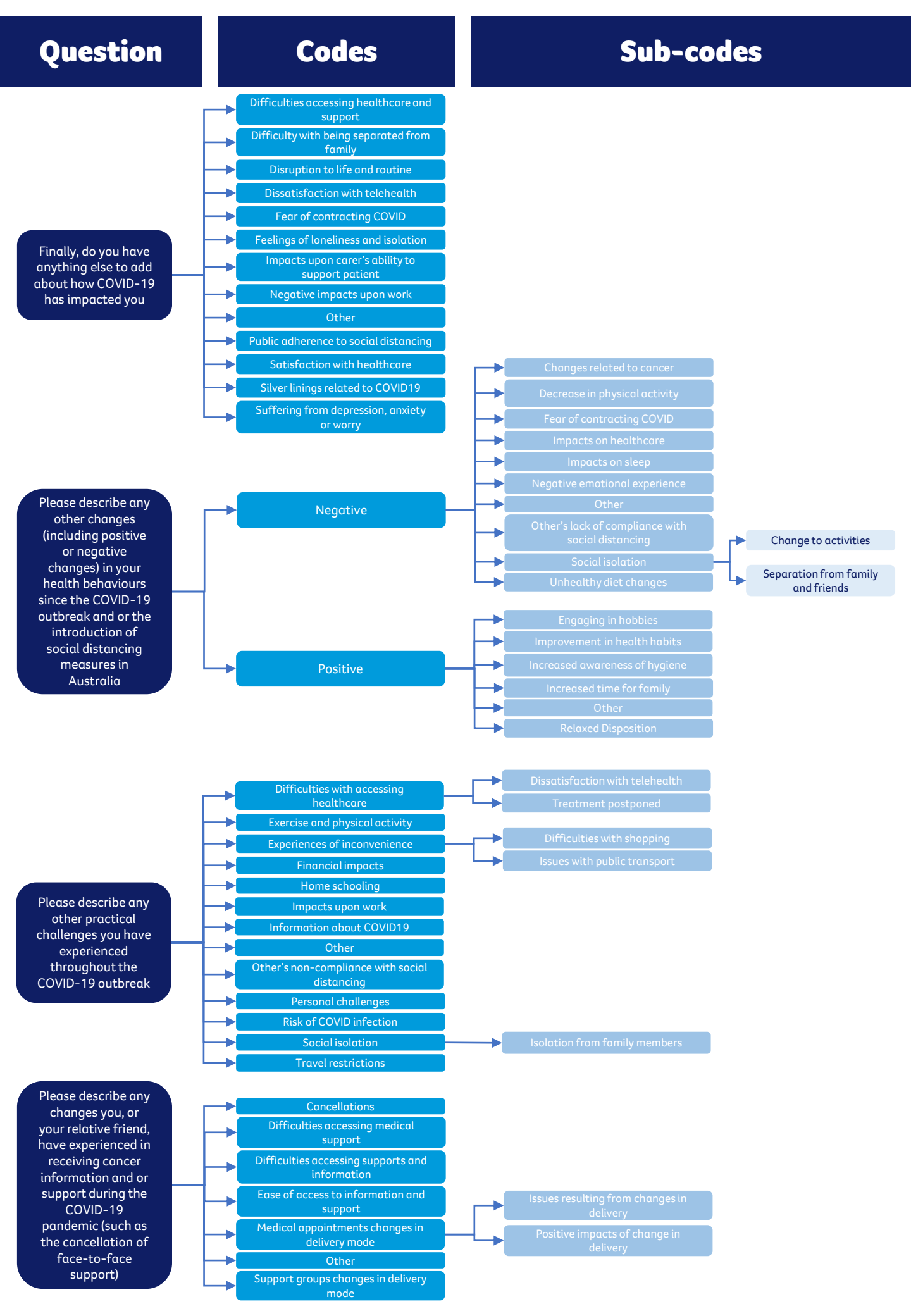

Supplement: S1 Survey — (PDF) [file pone.0257420.s001.pdf]

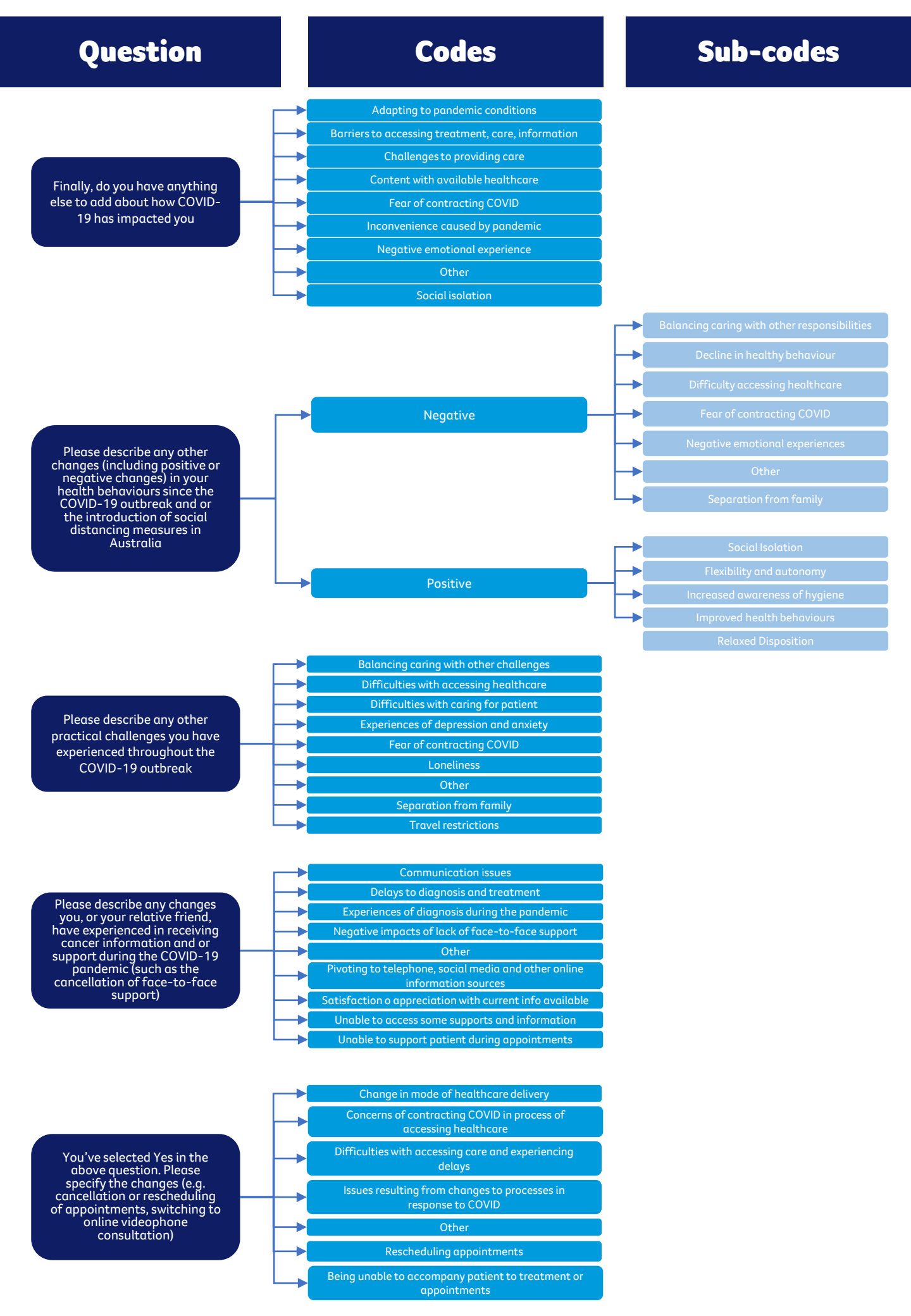

Supplement: S2 Survey — (PDF) [file pone.0257420.s002.pdf]
