# Supplementary material for: Cancer care disruption and reorganisation during the COVID-19 pandemic in Australia: A patient, carer and healthcare worker perspective
Source: PLoS One. 2021 Sep 17;16(9):e0257420. doi: 10.1371/journal.pone.0257420 (PMC8448370; doi:10.1371/journal.pone.0257420)
Supplement: S3 Survey — (DOCX) [file pone.0257420.s003.docx]

**Study 2 Appendix D_ Survey questions for healthcare workers**

**Please note, a draft version of this online survey is available here:**

<https://forms.office.com/FormsPro/Pages/ResponsePage.aspx?id=9FuR0UYVkEm81KvbA7iXA0LN2OkaR5xDpjO3tcl1DR5UM1NCTVFLMDZDTFVSTThaR09FRkRCTzZZOC4u>

If you are unable to finish responding to all the questions, please scroll to the end of the questionnaire and click submit

Questions marked with a * cannot be skipped

**SURVEY SCREENING QUESTIONS AND CONSENT**

Having read the participant information statement, do you consent to participate in this survey?*

- Yes
- No (Terminate survey)

Are you over the age of 18?*

- Yes
- No (Terminate survey)

Are you, or have you recently been, a healthcare worker involved in cancer care service delivery within Australia?*

- Yes
- No (Terminate survey)

**SECTION 1: DEMOGRAPHIC DATA**

1. What is your professional role?*

- Medical Doctor (other than a GP)
- GP
- Nurse
- Clinical trial coordinator
- Allied Health Professional
- Scientific staff (e.g. laboratory scientist)
- Administrative staff
- Ancillary staff (e.g. cleaner, porter)
- Other (include text field)

1. In your current (or most recent) professional role do you have **direct clinical interactions** with patients?*

- Yes (complete sections 2, 3, 4 (all), 5, 6)
- No (complete sections 2, 4 (half), 5, 6)

1. What is your age?

- 18-24 years
- 25-34 years
- 35-44 years
- 45-54 years
- 55-64 year
- 65-74 years
- 75-84 years
- 85-94 years
- 95 years and over

1. Which of the following best describes your gender?:

- Woman
- Man
- Non-binary
- Other:
- Prefer not to say

1. Approximately how long have you been practicing in your professional role?

- Less than 2 years
- 2- 5 years
- 6-10 years
- 11 – 20 years
- More than 20 years

1. Which is your primary work setting?*

- Inpatient public hospital
- Inpatient private hospital
- Speciality/dedicated cancer treatment centres (e.g. Chris O’Brien Lifehouse, Peter MacCallum Cancer Centre)
- Outpatient service
- Community care
- Private practice (e.g. private consulting rooms)
- Medical Centre / GP
- Other:

1. Is your primary work setting in?:

- Metropolitan area
- Rural area
- Remote area

1. In which state is your primary workplace location?:

- NSW
- ACT
- VIC
- QLD
- SA
- WA
- NT
- TAS

1. Do you work with a particular cancer type? Please select all that apply

- Brain and Spinal Cord
- Breast
- Colorectal
- Gynaecological
- Haematological
- Head and Neck
- Lung and Mesothelioma
- Palliative Care Service
- Pancreatic and Hepatobiliary
- Sarcoma
- Skin Cancer
- Upper Gastrointestinal
- Urological
- Any/all cancers
- Other

**Section 2: Preparedness for crisis and organisational resilience^[[1]](#footnote-1)^**

1. Please indicate how much you agree or disagree with the following statements in relation to your primary work setting taking into account the recent COVID-19 outbreak.

*(Strongly disagree – 0; Disagree – 1; Neutral – 2; Agree – 3; Strongly agree – 4)*

**Adaptive capacity**

There is a sense of teamwork and camaraderie in our organisation

Our organisation maintains sufficient resources to absorb some unexpected change

People in our organisation ‘‘own’’ a problem until it is resolved

Staff have the information and knowledge they need to respond to unexpected problems

Managers in our organisation lead by example

Staff are rewarded for ‘‘thinking outside the box’’

Our organisation can make tough decisions quickly

Managers actively listen for problems

**SECTION 3: CANCER CARE DELIVERY**

1. For the following questions, please indicate how much you agree or disagree with the following statements as they apply to you and your primary work role **since the start of the Australian outbreak of COVID-19** (approximately February 2020)*.*

*(Strongly disagree – 0; Disagree – 1; Neutral – 2; Agree – 3; Strongly agree – 4; N/A)*

1. I have had to change at least one aspect of my approach to care because of this crisis (if disagree or strongly disagree, skip remaining questions)

**Quality of care**

I am happy with the quality of care I have been able to provide to cancer patients during this crisis

Patients have been able to adhere to my prescribed treatment/care plan during the crisis

I have been able to discuss relevant patient cases with a multidisciplinary team as I would have before the crisis

Changes to cancer diagnostic procedures and/or pathways have made them suboptimal

Changes to treatment plans have made them suboptimal

There have been atypical delays in delivering care

Patient access to research and/or clinical trials has been reduced

I have been able to answer questions from cancer patients about the impact of COVID19

It has been more challenging than usual to involve caregivers in consults and appointments

Complexity of care has increased

I am concerned about the increased pressure COVID-19 has placed on patients’ mental health and wellbeing

**Time and priorities**

I had less time than usual during consults to discuss all relevant issues and concerns

I have been able to maintain continuing professional development during the crisis

I have had to allocate more time to procedures due to protective equipment and set up

I have been required to attend too many additional meetings because of the crisis

The additional number of meetings I have needed to attend due to the crisis have been worthwhile

**Resources**

The number of patient appointments I have been able to schedule has decreased from usual

The number of consultations cancelled by patients have increased from usual

I have been involved in more duties that take me away from patient consultations than usual (for example teaching and training of newly deployed staff, rapid research)

**Organisational support**

I have received adequate support to adopt alternative modes of service delivery (e.g. telehealth) to conduct patient appointments

I have been adequately informed about changes to clinical practice guidelines that affect my work

I have sufficient resources (pamphlets, brochures, websites) to which I can direct patients to learn more about how COVID-19 may impact their health

**Other**

1. Is there anything else you would like to say about your experience of cancer care delivery during this crisis event? (free text field)
2. Can you tell us approximately, at the peak of the crisis, what proportion of your appointments moved to telehealth?

- None
- A quarter
- Half
- More than half
- All

**SECTION 4: POSITIVE CHANGES TO HEALTHCARE DELIVERY**

1. I have noticed the following positive changes to my role **since the start of the Australian outbreak of COVID-19** (approximately February 2020)*.*

*(Strongly disagree – 0; Disagree – 1; Neutral – 2; Agree – 3; Strongly agree – 4; N/A)*

1. I am able to focus on things that are more important
2. I have become more efficient
3. I have improved my skills with technology
4. I have collaborated more with people within my organisation
5. I have collaborated more with people outside of my organisation
6. I have identified things about my own practice that were a waste of time
7. Staff have been able to flexibly adapt to new settings or practices
8. ￼I have noticed the following positive changes to healthcare delivery in my organisation **since the start of the Australian outbreak of COVID-19** (approximately February 2020)*.*

*(Strongly disagree – 0; Disagree – 1; Neutral – 2; Agree – 3; Strongly agree – 4; N/A)*

1. Technological changes in patient care delivery have allowed for more efficient patient management
2. Changes to rebate criteria have allowed for increased use of telehealth services
3. Patients and/or carers have increased capacity for self-management
4. Patients are less likely to pursue aggressive treatment
5. Patient and/or carer competency with telehealth has improved
6. Healthcare worker competency with telehealth has improved
7. Having to prioritise the most important things has highlighted work practices that are not needed
8. Rural services can cater better for cancer patients than previously recognised

**SECTION 5: RESPONSE AND RECOVERY STRATEGIES NEEDED**

1. Please select **up to 3 key areas** that you believe should be prioritised to support recovery from COVID-19 in Australia, or better prepare for crises that would affect cancer care in the future.

- Better planning for cancelled appointments
- Improved consistency of advice given during a crisis
- More training in delivering telehealth
- Better strategies to disseminate information effectively about a crisis
- Support to enable better communication with other healthcare workers about patient management during a crisis
- Improved safety resources (e.g., personal protective equipment, hand sanitiser, crowding control) should be provided by my organisation
- Practicing emergency plans in advance to help prepare for a future crisis
- Building/strengthening relationships with other organisations we might have to work with in a crisis
- Developing approaches to be able to shift rapidly from business as usual to crisis responses
- Clearer guidance is needed on phased return to usual practice

1. For each of your selections above, do you have any specific ideas to include?
2. Is there anything else you would like to say about response and recovery strategies for this or a similar crisis?

**SECTION 6: IMPACT ON YOU**

This question asks you about “moral distress”. Moral distress occurs when you believe you know the ethically correct thing to do, but something or someone restricts your ability to pursue the right course of action.

1. Please select the number that best describes how much moral distress you have been experiencing related to work **over the last week**.

*(None – 0; Mild – 2; Uncomfortable – 4; Distressing – 6; Intense – 8; Worst possible – 10)*

1. Please indicate how much you agree with the following statements as they apply to you **over the last week**.

*(Not at all – 0; Rarely True – 1; Sometimes True – 2; Often True – 4; True Nearly All the Time – 4)*

1. I am able to adapt when changes occur.
2. I can deal with whatever comes my way.
3. I try to see the humorous side of things when I am faced with problems.
4. Having to cope with stress can make me stronger.
5. I tend to bounce back after illness, injury, or other hardships.
6. I believe I can achieve my goals, even if there are obstacles.
7. Under pressure, I stay focused and think clearly.
8. I am not easily discouraged by failure.
9. I think of myself as a strong person when dealing with life’s challenges and difficulties.
10. I am able to handle unpleasant or painful feelings like sadness, fear, and anger.
11. This question asks you about your feelings and thoughts **during the last week**.

For each item, please indicate how often you felt or thought a certain way.

*(Never – 0; Almost never – 1; Sometimes – 2; Fairly often – 3; Very often – 4)*

1. In the last week, how often have you been upset because of something that happened unexpectedly?
2. In the last week, how often have you felt that you were unable to control the important things in your life?
3. In the last week, how often have you felt nervous and stressed?
4. In the last week, how often have you felt confident about your ability to handle your personal problems?
5. In the last week, how often have you felt that things were going your way?
6. In the last week, how often have you found that you could not cope with all the things that you had to do?
7. In the last week, how often have you been able to control irritations in your life?
8. In the last week, how often have you felt that you were on top of things?
9. In the last week, how often have you been angered because of things that happened that were outside of your control?
10. In the last week, how often have you felt difficulties were piling up so high that you could not overcome them?
11. This question asks you to think about what aspects of your work or home life are particularly stressful.

For each item, please select the number that best describes how much stress you have been experiencing.

*(0 = Not at all stressful, 10 = A major cause of stress)*

1. How much stress comes from your daily work environment (e.g., facilities, colleagues, non-clinical duties)?
2. How much stress comes from providing patient care?
3. How much stress comes from your risk of becoming infected with COVID-19?
4. How much stress comes from your concerns of transmitting COVID-19 to others?
5. How much stress comes from the need for social isolation?
6. How much stress comes from personal or family financial concerns?
7. How much stress comes from having children home from school?
8. Is there anything else that you would like to say about COVID-19 in the context of cancer care and recovery strategies?

**Thank you for taking part in this survey, your response is greatly valued.**

If you would like to receive a summary of the results, you can email the research team at [birde@nswcc.org.au](mailto:birde@nswcc.org.au) with the subject heading [TBC] and they will add you to a mailing list.

Do you consent to being contacted in the future for follow up about the aggregate findings of this study? This may be in the form of another survey or qualitative interview, subject to relevant ethics approval. You may withdraw your consent for this at any time by contacting [birde@nswcc.org.au](mailto:birde@nswcc.org.au).

**If you click yes you will be directed to another form to collect your name and email, these identifying details cannot be linked to your responses to this questionnaire.**

- Yes – direct to a separate webpage
- No - (end of survey)

1. Questions for this section are adapted from the Short-form version of the Benchmark Resilience Tool (BRT-53). The short version of BRT-53 is a validated tool for measuring organizational-level resilience. [↑](#footnote-ref-1)
