# Supplementary material for: Cancer care disruption and reorganisation during the COVID-19 pandemic in Australia: A patient, carer and healthcare worker perspective
Source: PLoS One. 2021 Sep 17;16(9):e0257420. doi: 10.1371/journal.pone.0257420 (PMC8448370; doi:10.1371/journal.pone.0257420)
Supplement: S4 Survey — (DOCX) [file pone.0257420.s004.docx]

**Study 1 Appendix D_Survey questions for people affected by cancer**

**Please note, a draft version of this online survey is available here^[[1]](#footnote-2)^:**

<https://forms.office.com/FormsPro/Pages/ResponsePage.aspx?id=9FuR0UYVkEm81KvbA7iXA0LN2OkaR5xDpjO3tcl1DR5UME1MNkMxQzJKOUdESjkzUUswQUNPWUw5MC4u>

If you are unable to finish responding to all the questions, please scroll to the end of the questionnaire and click submit

Questions marked with a * cannot be skipped

**SURVEY SCREENING QUESTIONS AND CONSENT**

Having read the participant information statement, do you consent to participate in this survey?*

 Yes

 No (Terminate survey)

Are you at least 18 years of age **and** currently living in Australia?*

 Yes

 No (Terminate survey)

Please describe how you are affected by cancer?*

 I have a current cancer diagnosis (Route to patient survey)

 I have been diagnosed with cancer in the past and am now in recovery or remission (Route to patient survey)

 I am a carer, family member, partner or friend of someone affected by cancer (Route to carer survey)

 None of the above (Terminate survey)

**SECTION 1: ABOUT YOU**

1. What is your age?*

 18-24 years

 25-34 years

 35-44 years

 45-54 years

 55-64 year

 65-74 years

 75-84 years

 85-94 years

 95 years and over

1. Which of the following best describes your gender?*

 Woman

 Man

 Non-binary

 Other: ______________

 Prefer not to say

1. How would you describe your ethnicity? (dropdown list)*

 Australian

 New Zealander

 Asian

 Indian

 Middle Eastern

 European

 North American

 South American

 African

 Prefer not to answer

 Other: ____________________________

1. Do you live in:*

 Metropolitan area

 Rural area

 Remote area

1. In which state or territory do you live?*

 NSW

 ACT

 VIC

 QLD

 SA

 WA

 NT

 TAS

- 1. What is the highest level of education that you have completed?

ð Less than Year 12 or equivalent

ð Year 12 or equivalent (HSC/Leaving cert)

ð Advanced diploma/diploma/Certificate III/IV

ð Bachelor degree

ð Postgraduate degree

- 1. How many people are currently living in your household? (dropdown of numbers)
  2. Have you, or anyone in your family been diagnosed with COVID-19?

ð Yes

ð No

- 1. Do you have any of the following health conditions? (Please select all that apply)

ð Diabetes

ð Heart Disease

ð Arthritis

ð Hypertension

ð Asthma or respiratory illness

ð Mental health conditions (e.g. depression, anxiety)

ð High cholesterol

ð Overweight or obesity

ð None of the above

**Questions for cancer patients and survivors**

**SECTION 2: CANCER DIAGNOSIS**

- 1. In what year were you diagnosed with cancer *(if you have been diagnosed with cancer on more than one occasion then please select the year of your most recent diagnosis)?* (dropdown list of years)
  2. What type(s) of cancer do you have/have you had? (Please select all that apply)

 Colorectal

 Prostate

 Breast

 Melanoma of the skin

 Lung

 Uterus

 Head and neck (including brain cancer)

 Lymphoma

 Thyroid

 Pancreas

 Kidney

 Leukaemia

 Bladder

 Liver

 Ovary

 Other

- 1. What stage would you describe your cancer?

 Early

 Localised

 Regional spread

 Distant spread

 Recovery or remission

 Unsure

 Not applicable

- 1. What treatment are you currently receiving or is pending for your cancer? (Please select all that apply)

 Chemotherapy

 Radiation therapy

 Anti-hormone

 Immunotherapy

 Surgery

 I am under active surveillance

 I am receiving palliative care

 I am participating or due to participate in a clinical trial

 I have completed treatment/not currently receiving treatment

 Other (please specify): ____________________________________________________

**SECTION 3: COVID-19 IMPACT ON EMOTIONAL WELLBEING**

In this section of the survey, we will ask you some questions about **your emotional wellbeing during the recent COVID-19 outbreak**.

- 1. Please select the number (0 – 10; 0 for no distress at all and 10 for extreme distress) that best describes how much distress you have been experiencing:
     - 1. ***[timeframe TBC] before the COVID-19 outbreak in Australia?***
       2. [***timeframe TBC] since the COVID-19 outbreak in Australia?***

1. In relation to COVID-19^[[2]](#footnote-3)^ , over the last two weeks, how often have you been bothered by:

|  | Not at all | Several days | More than half the days | Nearly every day |
| --- | --- | --- | --- | --- |
| feeling nervous, anxious or on edge? |  |  |  |  |
| not being able to stop or control worrying? |  |  |  |  |
| little interest or pleasure in doing things? |  |  |  |  |
| feeling down, depressed, or hopeless? |  |  |  |  |

1. The next questions are about how you feel about different aspects of your life in relation to the COVID-19 outbreak.^[[3]](#footnote-4)^

|  | **Before social distancing measures were introduced in March 2020** | | | **Since social distancing measures were introduced in March 2020** | | |
| --- | --- | --- | --- | --- | --- | --- |
| How often do you experience the following? | Hardly ever | Some of the time | Often | Hardly ever | Some of the time | Often |
| I lack companionship |  |  |  |  |  |  |
| I feel left out |  |  |  |  |  |  |
| I feel isolated |  |  |  |  |  |  |

1. The following concerns have been commonly reported during the COVID-19 outbreak. To what extent had each of them affected your thoughts and feelings?

|  | Insignificant | Minor | Moderate | Major | Severe | NA |
| --- | --- | --- | --- | --- | --- | --- |
| Risk of exposure to the virus and infection | ð | ð | ð | ð | ð | ð |
| Anxiety over your immunity to the virus | ð | ð | ð | ð | ð | ð |
| Fear of going to hospital or attending medical appointments | ð | ð | ð | ð | ð | ð |
| Concerns about going to work | ð | ð | ð | ð | ð | ð |
| Fear of infecting others in the household | ð | ð | ð | ð | ð | ð |

**SECTION 4: PRACTICAL NEEDS**

In this section of the survey, we will ask you some questions about your financial situation and your practical needs.

1. What was your employment situation before the COVID-19 outbreak?

ð Full-time

ð Part-time

ð Casual

ð Unemployed

ð Retired

1. Have you experienced any of the following changes to your employment and income situation as a result of the COVID-19 outbreak? (Please select all that apply)

ð Personal loss of employment or being stood down

ð Quit work or taken leave due to the risk of COVID-19

ð Reduced pay and/or work hours

ð Change of work arrangement (e.g. working from home)

ð Loss of employment or reduced income for members in your household

ð Received payments from JobKeeper/JobSeeker/other income support

ð No change experienced

ð Other, please specify _________________________________________

1. Thinking about your financial situation and the impact of the COVID-19 outbreak, on the scale of 0 to 10, how would you rate the level of your financial stress?

|  | 0 | 1 | 2 | 3 | 4 | 5 | 6 | 7 | 8 | 9 | 10 |
| --- | --- | --- | --- | --- | --- | --- | --- | --- | --- | --- | --- |
|  | (No stress at all) | | | |  |  | (Overwhelming stress) | | | | |
| [timeframe TBC] **before** the COVID-19 outbreak in Australia | ð | ð | ð | ð | ð | ð | ð | ð | ð | ð | ð |
| [timeframe TBC] **since** the COVID-19 outbreak in Australia | ð | ð | ð | ð | ð | ð | ð | ð | ð | ð | ð |

1. Comparing to your household income prior to the COVID-19 outbreak, what is the estimated income loss your household has experienced in the [timeframe TBC]?

ð No income loss

ð 1-20%

ð 21-40%

ð 41-60%

ð 61-80%

ð More than 80%

ð Unsure

1. To what extent do you agree or disagree with the following statements in relation to your financial and employment situation?

| Due to the economic impact of COVID-19, as time goes on… | Strongly disagree | Disagree | Neither agree nor disagree | Agree | Strongly Agree | NA |
| --- | --- | --- | --- | --- | --- | --- |
| I feel more worried and anxious about my financial situation | ð | ð | ð | ð | ð | ð |
| I feel more worried and anxious about my ability to afford my cancer care and treatment | ð | ð | ð | ð | ð | ð |
| I feel more worried and anxious about my ability to afford living expenses such as groceries, transport, and rent | ð | ð | ð | ð | ð | ð |
| I have an increasing amount of debt | ð | ð | ð | ð | ð | ð |
| I am concerned that I won’t be able to return to work as planned | ð | ð | ð | ð | ð | ð |
| I am concerned about my leave entitlements at work | ð | ð | ð | ð | ð | ð |

1. To what extent have you experienced any of the following issues or practical challenges throughout the COVID-19 outbreak?

|  | No issue | Insignificant | Minor | Moderate | Major | Severe |
| --- | --- | --- | --- | --- | --- | --- |
| Difficulties with shopping and obtaining essential items (e.g. groceries, hand sanitiser, toilet paper) | ð | ð | ð | ð | ð | ð |
| Getting prescription and over-the-counter medication | ð | ð | ð | ð | ð | ð |
| Accessing transport to/from medical appointments | ð | ð | ð | ð | ð | ð |
| Obtaining information I need about cancer and COVID-19 | ð | ð | ð | ð | ð | ð |
| Accessing financial support packages and services | ð | ð | ð | ð | ð | ð |
| Staying physically active and doing regular exercise | ð | ð | ð | ð | ð | ð |

1. Please describe any other practical challenges you have experienced throughout the COVID-19 outbreak (free text comment box):

**SECTION 5: BEHAVIOURS**

In this section of the survey, we will ask you some questions about your health behaviours and whether these have changed during the COVID-19 outbreak.

1. Please indicate the extent to which you have changed the following behaviours…

| As a result of COVID-19 and social distancing measures in Australia: | Significantly decreased | Slightly decreased | Not changed | Slightly increased | Significantly increased |
| --- | --- | --- | --- | --- | --- |
| My alcohol intake has… | ð | ð | ð | ð | ð |
| My physical activity has… | ð | ð | ð | ð | ð |
| The quality of my diet has… | ð | ð | ð | ð | ð |
| My ability to follow the dietary recommendations from my doctor has… | ð | ð | ð | ð | ð |
| My tobacco use has… | ð | ð | ð | ð | ð |
| The frequency that I go to the doctor for routine check-ups and/or appointments (e.g. screening tests, vaccinations) has… | ð | ð | ð | ð | ð |
| The quality of my sleep has… | ð | ð | ð | ð | ð |

1. Please describe any other changes (including positive or negative changes) in your health behaviours since the COVID-19 outbreak and/or the introduction of social distancing measures in Australia (free text comment box):

**SECTION 6: CANCER CARE AND SERVICES**

In this section of the survey, we will ask about your experiences accessing treatment and cancer services throughout the COVID-19 outbreak.

1. a) Have you experienced any of the following changes to your cancer care since the COVID-19 outbreak in Australia?

|  | Cancelled | Rescheduled | No change | NA |
| --- | --- | --- | --- | --- |
| Medical appointments | ð | ð | ð | ð |
| Cancer treatment | ð | ð | ð | ð |
| Surgery or operation | ð | ð | ð | ð |
| Cancer screening or tests | ð | ð | ð | ð |
| Clinical trials | ð | ð | ð | ð |

b) (If selected any of the cancellation/postponement options) What were the reasons for the cancellation or postponement (please select all that apply)?

ð My healthcare provider cancelled or postponed without reason

ð My healthcare provider cancelled or postponed with good explanation of risks and benefits

ð My healthcare provider needed to switch to online/video/phone consultations

ð I cancelled or postponed because I was worried about the risk of COVID-19

ð I cancelled or postponed because I could not afford the cost

ð Other reasons ________________________________________________

1. The following questions ask about your cancer care experience during the COVID-19 outbreak.

|  | Strongly Disagree | Disagree | Neutral | Agree | Strongly Agree | NA |
| --- | --- | --- | --- | --- | --- | --- |
| I felt fully informed about my susceptibility to COVID-19 | ð | ð | ð | ð | ð | ð |
| I had access to practical and emotional support services that I needed (e.g. counselling, cancer support groups, nutritional advice) | ð | ð | ð | ð | ð | ð |
| I felt fully informed about changes to my treatment plan | ð | ð | ð | ð | ð | ð |
| I had been fully informed about my financial entitlements (eg Medicare and health fund claims, travel allowances etc) | ð | ð | ð | ð | ð | ð |
| I experienced longer wait times for an appointment (e.g. consultation, test or treatment) | ð | ð | ð | ð | ð | ð |
| I found it difficult to get an appointment with my regular healthcare provider (GP, specialist, allied health professional) | ð | ð | ð | ð | ð | ð |

1. Have you used any information and/or support services from Cancer Council:
2. **Before** the COVID-19 outbreak [timeframe TBC]?

ð Yes ð No ð Unsure

1. **Since** the COVID-19 outbreak [timeframe TBC]?

ð Yes ð No ð Unsure

1. Please describe any changes you have experienced in receiving cancer information and/or support during the COVID-19 pandemic? (such as the cancelation of face-to-face support groups, peer support over the phone or online)

_________________________________________________________________________________

**SECTION 7: TELEHEALTH SERVICES**

1. Have you had any experience of using telehealth services before the COVID-19 outbreak?

ð Yes

ð No

1. Since [timeframe TBC], have you had any consultations or medical appointments via telehealth services (i.e. phone, video or online consultations)?

ð Yes (continue to the next question)

ð No (direct to the question 32 and then 33)

1. With whom have these recent telehealth consultations been? (Please tick all that apply)

ð GP

ð Specialist consultant

ð Mental health professional

ð Allied Health Professional (for example a Physiotherapist or Dietitian)

ð Other (please specify) ________________________________________

1. Please rate your overall experience of the telehealth services you received:

|  | Poor | Fair | Average | Good | Excellent |
| --- | --- | --- | --- | --- | --- |
| Overall satisfaction of the appointment/consultation | ð | ð | ð | ð | ð |
| Accessibility and convenience of attending appointment | ð | ð | ð | ð | ð |
| Process of using the service (e.g. dialling or logging in) | ð | ð | ð | ð | ð |
| Quality of the care received | ð | ð | ð | ð | ð |
| Psychological and emotional support received | ð | ð | ð | ð | ð |
| Respect of privacy | ð | ð | ð | ð | ð |

1. Please tell us about any benefits you have experienced in using telehealth services (Please select all that apply):

ð Improved access to services

ð Reduced costs such as travel, parking, childcare

ð More timely services (e.g. shorter wait time for appointments)

ð More flexibility and less interruption to work or routines

ð Improved quality of care and services

ð Availability for a bulk-billed appointment

ð Reduced possible exposure to COVID-19 (e.g. at hospital, on public transport)

ð Other (please specify) _______________________________

1. Did you experience any of the following difficulties or issues when you used or tried to use telehealth services? (Please select all that apply)

ð Difficulties scheduling an appointment

ð Issues with internet access

ð Issues using the technology

ð I was not able to see my usual practitioner

ð Telehealth service was not bulk-billed

ð Concerns about privacy

ð Concerns about the quality of your care (e.g. not being able to have a physical examination)

ð None of the above

ð Not applicable

ð Other (please specify) _________________________________

1. How likely are you to use telehealth services in the future when [Timeframe TBC]?

ð Very likely

ð Likely

ð Neutral

ð Unlikely

ð Very Unlikely

**Questions for cancer carers, family members and friends**

**SECTION 2: CARER CHARACTERISTICS**

- 1. What is your relationship to the person living with cancer?

 Spouse or partner

 Child of the person living with cancer

 Parent of the person living with cancer

 Sibling

 Other

- 1. Do you live with the person diagnosed with cancer?

 Yes

 No

- 1. How often do you provide care or support for the person living with cancer (*including practical help with daily living such as transport and meal preparation; cancer care such as managing medication and accompanying to medical appointments; and psychological and emotional support*)?

 Daily

 Several time a week

 Several times a month

 Less than once a month (Skip the rest of Section 2 and Skip Section 6 and 7)

- 1. In what year was the person diagnosed with cancer *(if he/she has been diagnosed with cancer on more than one occasion then please select the year of the most recent diagnosis)?* (dropdown list of years)
  2. What type(s) of cancer does he/she have? (Please select all that apply)

 Colorectal

 Prostate

 Breast

 Melanoma of the skin

 Lung

 Uterus

 Head and neck (including brain cancer)

 Lymphoma

 Thyroid

 Pancreas

 Kidney

 Leukaemia

 Bladder

 Liver

 Ovary

 Other

- 1. What stage would you describe his/her cancer?

 Early

 Localised

 Regional spread

 Distant spread

 Recovery or remission

 Unsure

 Not applicable

- 1. What treatment is he/she currently receiving? (Please select all that apply)

 Chemotherapy

 Radiation therapy

 Anti-hormone

 Immunotherapy

 Surgery

 Under active surveillance

 Receiving palliative care

 Participating or due to participate in a clinical trial

 Completed treatment/not currently receiving treatment

 Other (please specify): ____________________________________________________

**SECTION 3: COVID-19 IMPACT ON EMOTIONAL WELLBEING**

In this section of the survey, we will ask you some questions about **your own** emotional wellbeing **during the COVID-19 outbreak**.

1. Please select the number (0 – 10; 0 for no distress and 10 for extreme distress) that best describes how much distress you have been experiencing:
   - - 1. [timeframe TBC] **before** the COVID-19 outbreak in Australia?
       2. [timeframe TBC] **since** the COVID-19 outbreak in Australia?
2. In relation to COVID-19^[[4]](#footnote-5)^, over the last two weeks, how often have you been bothered by:

|  | Not at all | Several days | More than half the days | Nearly every day |
| --- | --- | --- | --- | --- |
| feeling nervous, anxious or on edge? |  |  |  |  |
| not being able to stop or control worrying? |  |  |  |  |
| little interest or pleasure in doing things? |  |  |  |  |
| feeling down, depressed, or hopeless? |  |  |  |  |

1. The next questions are about how you feel about different aspects of your life in relation to the COVID-19 outbreak.^[[5]](#footnote-6)^

|  | **Before social distancing measures were introduced in March 2020** | | | **Since social distancing measures were introduced in March 2020** | | |
| --- | --- | --- | --- | --- | --- | --- |
| How often do you experience the following? | Hardly ever | Some of the time | Often | Hardly ever | Some of the time | Often |
| I lack companionship |  |  |  |  |  |  |
| I feel left out |  |  |  |  |  |  |
| I feel isolated |  |  |  |  |  |  |

1. The following concerns have been commonly reported during the COVID-19 outbreak. To what extent has each of them affected your thoughts and feelings?

|  | Insignificant | Minor | Moderate | Major | Severe | NA |
| --- | --- | --- | --- | --- | --- | --- |
| Risk of your relative’s or friend’s (who is living with cancer) exposure to the virus and infection | ð | ð | ð | ð | ð | ð |
| Anxiety over your relative’s or friend’s immunity to the virus | ð | ð | ð | ð | ð | ð |
| Fear of going or accompanying your relative or friend to hospital or attending medical appointments | ð | ð | ð | ð | ð | ð |
| Concerns about going to work | ð | ð | ð | ð | ð | ð |
| Fear of infecting others in the household | ð | ð | ð | ð | ð | ð |

**SECTION 4: PRACTICAL NEEDS**

In this section of the survey, we will ask you some questions about **your own** financial situation and practical needs.

1. What was your employment situation before the COVID-19 outbreak?

ð Full-time

ð Part-time

ð Casual

ð Unemployed

ð Retired

1. Have you experienced any of the following changes to your employment and income situation as a result of the COVID-19 outbreak? (Please select all that apply)

ð Personal loss of employment or being stood down

ð Quit work or took leave due to the risk of COVID-19 for the person living with cancer

ð Reduced pay and/or work hours

ð Change of work arrangement (e.g. working from home)

ð Loss of employment or reduced income for members in your household

ð Received payments from JobKeeper/JobSeeker/other income support

ð No change experienced

ð Other, please specify _________________________________________

1. Thinking about your financial situation and the impact of the COVID-19 outbreak, on the scale of 0 to 10, how would you rate the level of your financial stress?

|  | 0 | 1 | 2 | 3 | 4 | 5 | 6 | 7 | 8 | 9 | 10 |
| --- | --- | --- | --- | --- | --- | --- | --- | --- | --- | --- | --- |
|  | (No stress at all) | | | |  |  | (Overwhelming stress) | | | | |
| [timeframe TBC] **before** the COVID-19 outbreak in Australia | ð | ð | ð | ð | ð | ð | ð | ð | ð | ð | ð |
| [timeframe TBC] **since** the COVID-19 outbreak in Australia | ð | ð | ð | ð | ð | ð | ð | ð | ð | ð | ð |

1. Compared to your household income prior to the COVID-19 outbreak, what is the estimated income loss you have experienced in your household in the last month?

 No income loss

 1-20%

 21-40%

 41-60%

 61-80%

 More than 80%

 Unsure

1. To what extent do you agree or disagree with the following statements in relation to your financial situation?

| Due to the economic impact of COVID-19, as time goes on… | Strongly disagree | Disagree | Neither agree nor disagree | Agree | Strongly Agree | NA |
| --- | --- | --- | --- | --- | --- | --- |
| I feel more worried and anxious about my financial situation | ð | ð | ð | ð | ð | ð |
| I feel more worried and anxious about the cost associated with cancer care and treatment | ð | ð | ð | ð | ð | ð |
| I feel more worried and anxious about my ability to afford living expenses such as groceries, transport, and rent | ð | ð | ð | ð | ð | ð |
| I have an increasing amount of debt | ð | ð | ð | ð | ð | ð |
| I am concerned that I won’t be able to return to work as anticipated or planned | ð | ð | ð | ð | ð | ð |
| I am concerned about my leave entitlements at work | ð | ð | ð | ð | ð | ð |

1. To what extent have you experienced any of the following issues or practical challenges throughout the COVID-19 outbreak?

|  | No issue | Insignificant | Minor | Moderate | Major | Severe | NA |
| --- | --- | --- | --- | --- | --- | --- | --- |
| Difficulties with shopping and obtaining essential items (e.g. groceries, hand sanitiser, toilet paper) | ð | ð | ð | ð | ð | ð | ð |
| Getting prescription and over-the-counter medication | ð | ð | ð | ð | ð | ð | ð |
| Obtaining information about cancer and COVID-19 | ð | ð | ð | ð | ð | ð | ð |
| Accessing financial support packages and services | ð | ð | ð | ð | ð | ð | ð |
| Staying physically active and doing regular exercise | ð | ð | ð | ð | ð | ð | ð |
| Caring for and supporting the person living with cancer (e.g. attending medical appointments together, providing transport) | ð | ð | ð | ð | ð | ð | ð |
| Visiting the person living with cancer at their home | ð | ð | ð | ð | ð | ð | ð |
| Visiting the person living with cancer outside their home (e.g. hospital, treatment centre, hospice) | ð | ð | ð | ð | ð | ð | ð |
| Accessing respite care or short-term care | ð | ð | ð | ð | ð | ð | ð |
| Managing relationship with the person living with cancer (e.g. maintaining personal space during quarantine) | ð | ð | ð | ð | ð | ð | ð |

1. Please describe any other practical challenges you have experienced throughout the COVID-19 outbreak. (free text comment box)

**SECTION 5: BEHAVIOURS**

In this section of the survey, we will ask you some questions about **your own** health behaviours and whether these have changed during the COVID-19 outbreak.

1. Please indicate the extent to which you have changed the following behaviours…

| As a result of COVID-19 and social distancing measures in Australia: | Significantly decreased | Slightly decreased | Not changed | Slightly increased | Significantly increased |
| --- | --- | --- | --- | --- | --- |
| My alcohol intake has… | ð | ð | ð | ð | ð |
| My physical activity has… | ð | ð | ð | ð | ð |
| The quality of my diet has… | ð | ð | ð | ð | ð |
| My ability to follow the dietary recommendations from my doctor has… | ð | ð | ð | ð | ð |
| My tobacco use has… | ð | ð | ð | ð | ð |
| The frequency that I go to the doctor for issues that could be postponed (e.g. cancer screening, vaccination, check-ups) has… | ð | ð | ð | ð | ð |
| The quality of my sleep has… | ð | ð | ð | ð | ð |

1. Please describe any other changes (including positive or negative changes) in your health behaviours since the COVID-19 outbreak and/or the introduction of social distancing measures in Australia (free text comment box):

**SECTION 6: CANCER CARE AND SERVICES**

In this section of the survey, we want to know about you and/or your relative’s or friend’s experience in accessing cancer treatment and support services during the COVID-19 outbreak. For each question, please reflect on the experience of the person living with cancer. If you do not feel comfortable with providing answers to any of the questions in this section, please feel free leave them blank and skip to the next question.

1. a) Have there been any changes to the way your relative or friend receive cancer care since the COVID-19 outbreak in Australia?

|  | Cancelled | Rescheduled | No change | NA |
| --- | --- | --- | --- | --- |
| Medical appointments | ð | ð | ð | ð |
| Cancer treatment | ð | ð | ð | ð |
| Surgery or operation | ð | ð | ð | ð |
| Cancer screening or tests | ð | ð | ð | ð |
| Clinical trials | ð | ð | ð | ð |

b) (If selected any of the cancellation/postponement options) What were the reasons for the cancellation or postponement (please select all that apply)?

ð The healthcare provider cancelled or postponed without reason

ð The healthcare provider cancelled or postponed with good explanation of risk and benefits

ð The healthcare provider needed to switch to online/video/phone consultations

ð He/she/we cancelled or postponed due to concerns about the risk of COVID-19

ð He/she/we cancelled or postponed because he/she/we could not afford the cost

ð Other reasons ________________________________________________

1. The following questions ask about your relative’s or friend’s cancer care experience during the COVID-19 outbreak.

|  | Strongly Disagree | Disagree | Neutral | Agree | Strongly Agree | NA |
| --- | --- | --- | --- | --- | --- | --- |
| He/she/we felt fully informed about the person’s (person living with cancer) susceptibility to COVID-19 | ð | ð | ð | ð | ð | ð |
| He/she/we had access to the practical and emotional support services needed (e.g. counselling, cancer support groups, nutritional advice) | ð | ð | ð | ð | ð | ð |
| He/she/we felt fully informed about changes to their treatment plan | ð | ð | ð | ð | ð | ð |
| He/she/we was/were fully informed about financial entitlements (e.g. Medicare and health fund claims, travel allowances etc) | ð | ð | ð | ð | ð | ð |
| He/she/we had experienced longer wait time for an available appointment (e.g. consultation, test or treatment) | ð | ð | ð | ð | ð | ð |
| He/she/we found it difficult to get an appointment with their regular healthcare provider (GP, specialist, allied health professional) | ð | ð | ð | ð | ð | ð |

1. Have you, or your relative/friend, used any information and/or support services from Cancer Council:
2. **Before** the COVID-19 outbreak [timeframe TBC]?

ð Yes ð No ð Unsure

1. **Since** the COVID-19 outbreak [timeframe TBC]?

ð Yes ð No ð Unsure

1. Please describe any changes you, or your relative/friend, have experienced in receiving cancer information and/or support during the COVID-19 pandemic? (such as the cancelation of face-to-face support groups, peer support over the phone or online)

_________________________________________________________________________________

**SECTION 7: TELEHEALTH SERVICES**

This section of the survey is about experience in accessing cancer treatment and support services via telehealth. For each question, please reflect on the experience of the person living with cancer. If you do not feel comfortable with providing answers to any of the questions in this section, please feel free to leave them blank and skip to the next question.

1. Have you had any experience of using telehealth services **before** the COVID-19 outbreak?

ð Yes

ð No

1. Since [timeframe TBC], has he/she had any consultations or medical appointments via telehealth services (i.e. phone, video or online consultations)?

ð Yes (continue to the next question)

ð No (direct to the next question 35 and then 36)

ð Unsure (direct to next question 35 and 36)

1. With whom have these recent telehealth consultations been with? (Please tick all that apply)

ð GP

ð Specialist consultant

ð Mental health professional

ð Allied health Professional (for example a Physiotherapist or Dietitian)

ð Other (please specify) ________________________________________

1. On behalf of the person living with cancer, please rate the overall experience of the telehealth services he/she received.

|  | Poor | Fair | Average | Good | Excellent |
| --- | --- | --- | --- | --- | --- |
| General satisfaction of the appointment/consultation | ð | ð | ð | ð | ð |
| Accessibility and convenience of attending appointment | ð | ð | ð | ð | ð |
| Process of using the service (e.g. dialling or logging in) | ð | ð | ð | ð | ð |
| Quality of the care received | ð | ð | ð | ð | ð |
| Psychological and emotional support received | ð | ð | ð | ð | ð |
| Respect of privacy | ð | ð | ð | ð | ð |

1. Please tell us about any benefits experienced in using telehealth services for the person with cancer (Please select all that apply):

ð Improved access to services

ð Reduced costs such as travel, parking, childcare

ð More timely services (e.g. shorter wait time for appointments)

ð More flexibility and less interruption to work or routines

ð Improved quality of care and services

ð Availability for a bulk-billed appointment

ð Reduced possible exposure to COVID-19 (e.g. at hospital, on public transport)

ð Other (please specify) _____________________________________________

1. Did he/she experience any of the following difficulties or issues in using or trying to use telehealth services? (Please select all that apply)

ð Difficulties scheduling an appointment

ð Issues with internet access

ð Issues using the technology

ð Not able to see his/her usual practitioner

ð Telehealth service was not bulk-billed

ð Concerns about privacy

ð Concerns about the quality of care e.g. from not being able to have a physical examination

ð None of the above

ð Not applicable

ð Other (please specify) ___________________________________________

1. How likely do you think he/she will use telehealth services in the future when [Timeframe TBC]?

ð Very likely

ð Likely

ð Neutral

ð Unlikely

ð Very Unlikely

**END OF SURVEY FOR PEOPLE AFFECTED BY CANCER (PATIENTS/SURVIVORS AND CARERS/FAMILIES/FRIEND)**

Finally, do you have anything else to add about how COVID-19 has impacted you? (free text)

Thank you for taking part in this survey, your response is greatly valued.

Do you consent to being contacted in the future for follow up about the aggregate findings of this study? This may be in the form of another survey or qualitative interview, subject to relevant ethics approval. You may withdraw your consent for this at any time by contacting birde@nswcc.org.au.

***If you click yes you will be directed to another form to collect your name and email, these identifying details cannot be linked to your responses to this questionnaire.***

ð Yes – direct to a separate webpage

ð No – end of survey

1. Please note that the online survey is a working draft, not all questions have been finalised yet on the online survey platform and some of the carer questions (Section 1, Q6-9) are yet to be included [↑](#footnote-ref-2)
2. The Patient Health Questionnaire-4 (PHQ-4) is a validated tool for measuring depression and anxiety. [↑](#footnote-ref-3)
3. The UCLA Loneliness – 3 items is a validated tool for measuring loneliness and perceived social support. [↑](#footnote-ref-4)
4. The Patient Health Questionnaire-4 (PHQ-4) is a validated tool for measuring depression and anxiety. [↑](#footnote-ref-5)
5. The UCLA Loneliness – 3 items is a validated tool for measuring loneliness and perceived social support. [↑](#footnote-ref-6)
